# High purity high yield tandem B and T helper cell isolation for qRT-PCR analysis suitable for basically equipped laboratories

**DOI:** 10.1186/s12936-018-2547-3

**Published:** 2018-10-29

**Authors:** Andrea Maria Summerauer, Lorenzo Colombo, Rodney Ogwang, Christoph Berger, Jan Fehr, Simone Bürgler

**Affiliations:** 1 Experimental Infectious Diseases and Cancer Research, Children’s Research Center, University Children’s Hospital Zurich, Zurich, Switzerland; 2 Makerere University, College of Health Sciences, Kampala, Uganda; 3 Centre of Tropical Neuroscience, Kitgum Site, Uganda; 4 Division of Infectious Diseases and Hospital Epidemiology, University Hospital Zurich, University of Zurich, Zurich, Switzerland; 5 Department of Public Health, Epidemiology Biostatistics and Prevention Institute, University of Zurich, Zurich, Switzerland

**Keywords:** B and T cell isolation from whole blood, High purity, Storage, Research in resource-limited setting, Basic equipment, Malaria, qRT-PCR analysis

## Abstract

**Background:**

Malaria is still a major health problem in sub-Saharan Africa and south-east Asia, but research on malaria in low-income countries can be a challenge due to the lack of laboratory equipment. In addition, severe malaria mainly affects very young children, which limits the amount of blood available for research purposes. Thus, there is a need for protocols that yield a maximum of information from a minimum amount of blood, which are operable in basically equipped laboratories.

**Results:**

A protocol for tandem B and T helper (Th) cell isolation directly from whole blood, and a freezer-independent sample preservation method compatible with the warm and humid climate of malaria regions was established and validated. The protocol thereby circumvents the need of high-technology centrifuges and unimpeachable power supply for peripheral blood mononuclear cell isolation. Both purity and yield are excellent. Depending on the expression level of the genes of interest, between 2 and 5 ml of blood are adequate for reliable qRT-PCR results from both B and Th cells of healthy paediatric donors as well as paediatric malaria patients.

**Conclusion:**

This protocol for high purity high yield B cell and Th cell isolation and sample storage for subsequent qRT-PCR analysis from a minimal amount of blood is contrivable with basic equipment and independent of continuous power supply. Thus, it is likely to be of avail for many scientists performing malaria research in rural institutes or hospitals, and thus in countries where malaria is most prevalent.

## Background

Malaria is still a major health problem in sub-Saharan Africa and south-east Asia. The World Health Organization (WHO) estimated 216 million new cases of malaria and 445,000 deaths in the year 2016 [1]. Even though treatment of severe malaria has improved during the last decade, prompt administration of effective drugs—a prerequisite to prevent multiple organ failure in severe malaria—remains a major challenge in rural areas with long distances from the closest health centre. In addition, several *Plasmodium* species develop resistance to anti-malarials [2]. Furthermore, in certain endemic areas such as equatorial Africa, patients that survive malaria have an increased risk of developing (and eventually dying from) Burkitt’s lymphoma [3]. Thus, development of therapeutic strategies that prevent rather than treat malaria—such as vaccines—are highly desirable. Unfortunately, anti-malaria vaccine development has turned out to be challenging. Even though natural infection in endemic areas results in immunity, this does not last indefinitely [4–6]. Furthermore, the immunity provided by natural infection seems to be very difficult to achieve using purified antigens [7]. It has been hypothesized that a malaria-related expansion of a certain B cell subset—referred to as “atypical” or “exhausted” B cells—may be a reason for the observed deficiency in the humoral response that hampers development of protective antibodies upon vaccination [8, 9]. The enzyme activation-induced cytidine deaminase (AID) plays a central role in class-switch recombination (CSR) and somatic hypermutation (SHM) [10]. AID expression in normal mature B cells within germinal centres is induced by T helper (Th)-cell derived signals such as CD40 ligation and cytokines [11]. Thus, for an efficient production of class-switched high-affinity antibodies, B cells depend on help from Th cells. Interestingly, a recent report provided evidence that not only B cells, but also Th cells may be dysfunctional in malaria patients [12]. Nevertheless, despite their importance in both malaria and anti-malaria vaccine development, very little is known about the phenotype and function of B and Th cells in malaria patients.

Performing malaria research in low-income countries—where malaria is most prevalent—is challenging and often hampered by the lack of equipment, unstable power supplies and absence of reliable cold-chains. In addition, severe malaria most often affects children under 5 years of age. Together with the fact that severe anaemia is one of the most common complication, this strictly limits the amount of blood available for research purpose, which hampers investigations on blood cells such as B and Th cells.

**Fig. 1:**
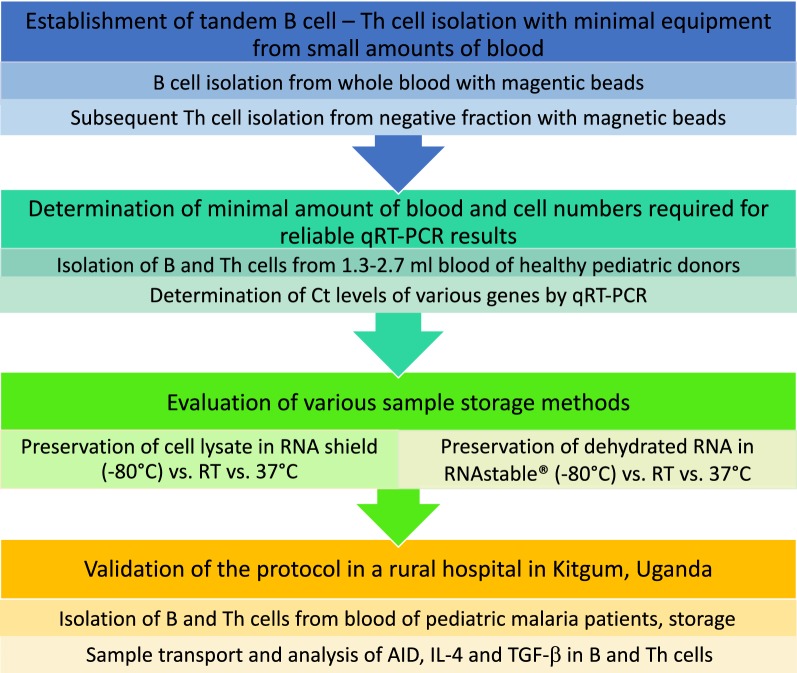
Establishment of the protocol. In a first step, tandem isolation of B cells and Th cells from whole blood was optimized and quality controlled for purity and efficiency by flow cytometry. Next, B cells and Th cells were isolated from small amounts of blood from healthy paediatric donors, cell numbers were determined and gene expression of various genes was analysed by qRT-PCR in order to determine the minimal amount of blood and cells necessary for reliable qRT-PCR results. Then, different preservation methods were compared under various conditions. Finally, the protocol was employed to analyse gene expression in B cells and Th cells from paediatric malaria patients isolated in a rural hospital in Uganda

The importance of understanding the development, nature and function of lymphocytes in malaria motivated us to develop a protocol for high purity, high yield B and Th cell isolation that is contrivable in basically equipped facilities and independent of high speed centrifuges or continuous power supply (Fig. 1). Depending on the expression levels of the genes of interest, 2–5 ml of blood is sufficient to isolate both B and Th cells, store the samples at room temperature (RT) for at least 1 month and analyse gene expression by conventional quantitative real-time polymerase chain reaction (qRT-PCR).

## Methods

### Healthy subjects

For establishment and validation of the tandem B and Th cells isolation protocol, cells were isolated from blood of adult volunteers, and healthy children undergoing routine tonsillectomy at the University Children’s Hospital Zurich. All experiments were previously approved by the regional ethics committee (Kantonale Ethikkommision Zürich, KEK).

### Malaria patients

Thirty four children meeting the clinical criteria for acute malaria [1] as well as having a positive result on a malaria rapid test (Binax NOW^®^), were enrolled at Kitgum general Hospital, Uganda after having obtained informed parental consent. All experiments were previously approved by the School of Medicine Research Ethics Committee, (SOMREC) as well as the Uganda National Council for Science and Technology (UNCST).

### Determination of parasite load

The parasite load of all individuals that were positive for malaria on HRP-2 RDT testing was determined according to standard protocols [1]. Briefly, thick blood smear slides were prepared and stained following standard Giemsa-staining protocols. Each slide was examined by two independent observers, and differences reconciled by a third observer. The number of asexual malaria parasites per 200 white blood cells (WBC) was reported and the parasite load per µl of blood estimated, assuming 8000 WBC/µl of blood.

### Blood sampling and serum preparation

Whole blood was collected in 9 ml Heparin Sarstedt Monovette tubes, following the common standard of care recommendations [13]. Serum was prepared by centrifuging 1 ml of whole blood at 1000*g* for 5 min.

### B and Th cell isolation from whole blood

The magnetic B and Th cell isolation from whole blood was carried out using the EasySep™ HLA Chimerism Whole Blood CD19 Positive Selection Kit (Stemcell Technologies) and the EasySep™ Human Whole Blood CD4 Positive Selection Kit (Stemcell Technologies) [14, 15]. The isolation medium was prepared by mixing 50 ml phosphate buffered saline (PBS) (BioConcept), 10 µl EDTA and 1 ml heat-inactivated FBS (Gibco). After several rounds of optimization, the following isolation procedure was established: The blood was collected in 9 ml Heparin tubes and transferred to 15 ml Falcon tubes for the isolation, adding a maximal amount of 4.5 ml blood per Falcon tube. First, the blood was mixed 1:1 with the Lysis buffer provided by the kit. Then, 25 µl per ml diluted blood sample of the B cell selection cocktail was added and samples were incubated for 5 min at RT. Next, 25 µl magnetic beads (rapid spheres) per ml diluted blood sample were added for 5 min. Afterwards, the sample volumes of ≤ 2.5 ml blood were topped up to 5 ml and samples volumes of > 5 ml were topped up to 10 ml with isolation medium. The diluted sample was incubated in the magnet without lid for 5 min. Importantly, instead of pouring off the supernatant as indicated in the manufacturer’s protocol, it was pipetted off with a 2 ml pipette, which dramatically increased the yield. This washing step was repeated twice, each time topping up the sample with 5 (or 10, respectively) ml of the isolation medium. Upon pipetting away the third supernatant, the falcon tube with the enriched B cells was removed from the magnet and the B cells were carefully eluted with complete RPMI (cRPMI) medium for counting and flow cytometry analysis.

Th cells were isolated from the first negative fraction after the B cell isolation, thus the red blood cells were already lysed at that time-point and the lysis step was omitted for the Th cell isolation. The other isolation steps were performed as follows: 25 µl of the CD4 selection cocktail per ml of diluted sample were added and the sample was incubated for 15 min. Thereafter, 25 µl magnetic nanoparticles per ml diluted samples were added and the sample was incubated for 10 min. To reduce the time of isolation process, the CD4 isolation was performed in parallel; i.e. the selection cocktail for the CD4 isolation was added to the negative fraction, when the B cell sample was on the magnet for the second washing step.

### Flow cytometry

To analyse the purity and efficiency of the B and Th cell isolation, flow cytometry analysis was performed. In order to identify B and Th cells, we stained for CD3 (total T cells), CD4 (Th cells) and CD19 (B cells) using fluorophore-conjugated antibodies (Table 1). To control for background signalling and to set the gates, isotype control antibodies were used. The cells were stained in staining buffer [PBS (BioConcept), 2% FBS (Gibco)] and subsequently fixed with 4% paraformaldehyde (Sigma) for 15 min. The cells were analysed with the BD FACSCanto ll flow cytometer (BD Bioscience). Data was processed by the BD FACSDiva software (BD Bioscience). The data was analysed using the FlowJo software (Treestar, Inc.).

**Table 1:** List of fluorophore-conjugated antibodies

Target	Fluorophore	Clone	Dilution	Company
CD4	FITC	OKT4	1:80	eBioscience
CD19	PE	HIB19	1:20	eBioscience
CD3	APC	UCHT1	1:80	Biolegend

### RNA extraction and determination of RNA integrity

To optimize sample storage, two different approaches were tested. On one side, RNA was extracted from the cells using the RNEasy Mini Kit (Qiagen) and then stored in RNAstable. On the other side, the cells were stored in RNAshield™ and subsequently, RNA was isolated using the Quick-RNA™ Micro Prep (Zymo Research). The concentration of RNA was measured with the NanoDrop™ 3300 Fluorospectrometer. To remove the remaining DNA, RNA was treated with DNAse (Ambion). RNA integrity was analysed using the Agilent Bioanalyzer 2100 System (Agilent technology).

### qRT-PCR

Reverse transcription was performed using the Applied Bioscience Kit. The mRNA expression of hydroxymethylbilane synthase HMBS, glyceraldehyde-3-phosphate dehydrogenase (G3PDH/GAPDH), 18sRNA [16–18]. CD19, AID, RAG1, IL-4 and TGF-b were analysed by qRT-PCR using the TaqMan^®^ technique with predesigned TaqMan^®^ Assays (Thermo Fisher Scientific).

### Diagrams and statistics

Data was analysed using the GraphPad Prism Version 7. For statistical significance, one- and two-way ANOVA tests were applied. For assessing correlations, Spearman test was used.

## Results

### High purity tandem B cell Th cell isolation from whole blood using magnetic beads

Often, malaria research needs to be performed in rural areas without access to modern laboratory equipment. To enable lymphocyte cell isolation in the absence of continuous power supply and high-speed centrifuges, a power-independent protocol accomplishable at RT needed to be established. Most methods for B cell and Th cell purification start with the isolation of PMBC using a density gradient [19]. Since this requires both a high-speed centrifuge and an unimpeachable continuous power supply, an approach where B and Th cells are isolated directly from whole blood using magnetic beads coupled to antibodies against surface antigens was chosen. Thus, kits starting from isolated peripheral blood mononuclear cell (PBMC) such as MagniSort™ Human CD19 Positive Selection Kit [20], MojoSort™ Human Pan B Cell Isolation Kit [21], Miltenyi Biotec B Cell Isolation Kit II, human [22] were excluded. As B cells are less abundant in blood than Th cells, isolation of B cells was prioritized and a positive isolation method that enables subsequent isolation of Th cells from the negative fraction was selected.

**Fig. 2:**
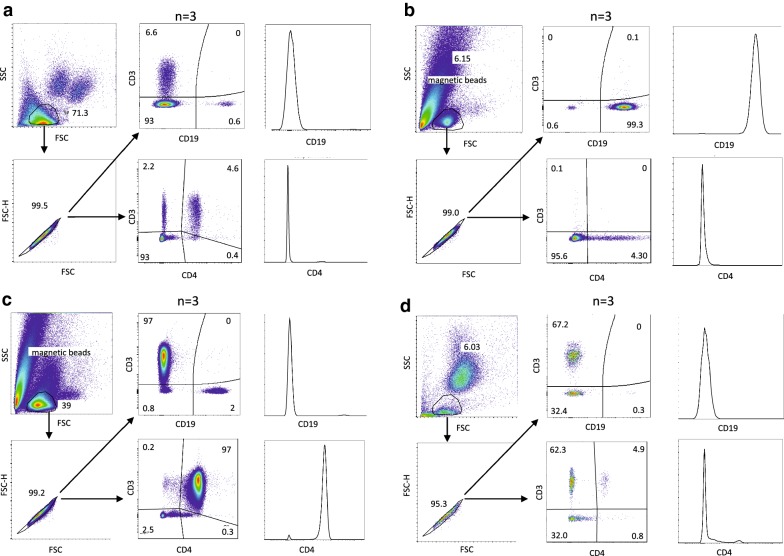
Flow cytometry analysis of purity and efficiency of B and Th cell isolation. Shown is a representative analysis of three independent isolations. **a** Flow cytometry analysis of whole blood after erythrocyte lysis (= before B cell isolation). Upper left: gating of live lymphocytes. Lower left: gating of single cells. Upper right: total T cells (CD3+) versus B cells (CD19+). Lower left: total T cells (CD3+) versus Th cells (CD3+CD4+). **b** Flow cytometry analysis of isolated CD19+ B cells (= positive fraction after B cell isolation). Upper left: gating of live lymphocytes. Lower left: gating of single cells. Upper middle: total T cells (CD3+) versus B cells (CD19+). Lower middle: total T cells (CD3+) versus Th cells (CD3+CD4+). Upper right: histogram of CD19 positive cells. Lower right: histogram of CD4 positive cells. **c** Flow cytometry analysis of isolated CD4+ Th cells (= positive fraction after Th cell isolation). Upper left: gating of live lymphocytes. Lower left: gating of single cells. Upper middle: total T cells (CD3+) versus B cells (CD19+). Lower middle: total T cells (CD3+) versus Th cells (CD3+CD4+). Upper right: histogram of CD19 positive cells. Lower right: histogram of CD4 positive cells. **d** Flow cytometry analysis of the negative fraction after Th cell isolation. Upper left: gating of live lymphocytes. Lower left: gating of single cells. Upper middle: total T cells (CD3+) versus B cells (CD19+). Lower middle: total T cells (CD3+) versus Th cells (CD3+CD4+). Upper right: histogram of CD19 positive cells. Lower right: histogram of CD4 positive cells

Thus, the final protocol was set up using the EasySep™ HLA Chimerism Whole Blood CD19 Positive Selection Kit (Stemcell Technologies) for B cell isolation [14] with a subsequent Th cell isolation using the EasySep™ Human Whole Blood CD4 Positive Selection Kit (Stemcell Technologies) [15]. To optimize and validate this isolation strategy, 5 ml of blood from healthy adult individuals were used. Purity and efficacy of the isolation process were assessed by flow cytometry (Fig. 2). After several rounds of optimization, a purity of 98.3 ± 2.3% for the B cells and 97.8 ± 3.1% for the Th cells, and a yield of 96.1 ± 3.0% for the B cells and 98.0 ± 2.2% for the Th cells was reached. Thus, both isolation steps achieved excellent purities and high yield, which is of great importance when the available blood volume is limited.

**Fig. 3:**
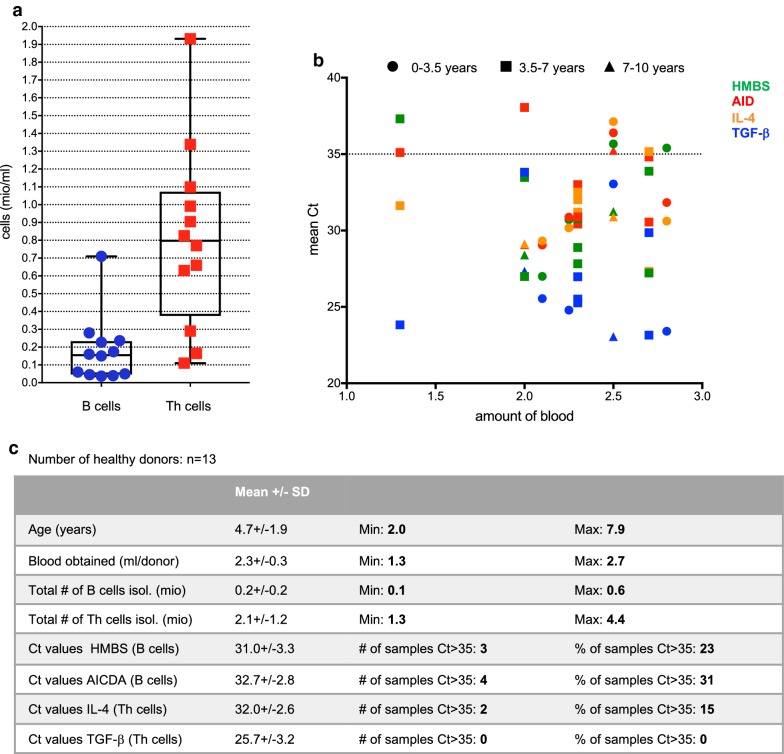
Cell number and Ct value analysis after B and Th cell isolation from whole blood of healthy paediatric donors. Blood was withdrawn from 13 healthy paediatric donors, B and Th cells were isolated and gene expression of AID, IL-4, TGF-b as well as the housekeeping gene HMBS was analysed by qRT-PCR. **a** B cell and Th cell numbers per ml of blood. **b** Amounts of blood withdrawn versus Ct values of HMBS (green), AID (red), IL-4 (orange) and TGF-b (blue). Symbol shapes indicate three different age groups. **c** Table summarizing parameters related to donors, B and Th cell isolation and gene expression analysis

Next, the number of B and Th cells that can be isolated from small amounts of blood was determined, and it was investigated, whether these numbers are high enough for reliable qRT-PCR analysis. To mimic the situation of paediatric malaria, 1.3–2.7 ml blood from 13 healthy children aged between 2 and 8 years was used to isolate B and Th cells with the established protocol (Fig. 3). In these experiments, a B cell count of 136,700/ml (95% CI [81,230–192,100]) and a Th cell count of 833,800/ml (95% CI 532,300–1,135,000]) was obtained (Fig. 3a). Expression of the housekeeping gene HBMS, but also of the B cell gene AID and the Th cell genes IL-4 and TGF-b was analysed by qRT-PCR. While Ct values for TGF-b were lower than 35 in all samples, the Ct values for AID were above 35 in almost one-third of the samples (Fig. 3b, c). Thus, for genes with high expression levels, 1.3–2.7 ml blood from healthy individuals is enough, while for other genes, more blood is required and/or some samples may need to be excluded from the analysis due to low reliability of the qRT-PCR results.

### Determination of the most suitable preservation method

**Fig. 4:**
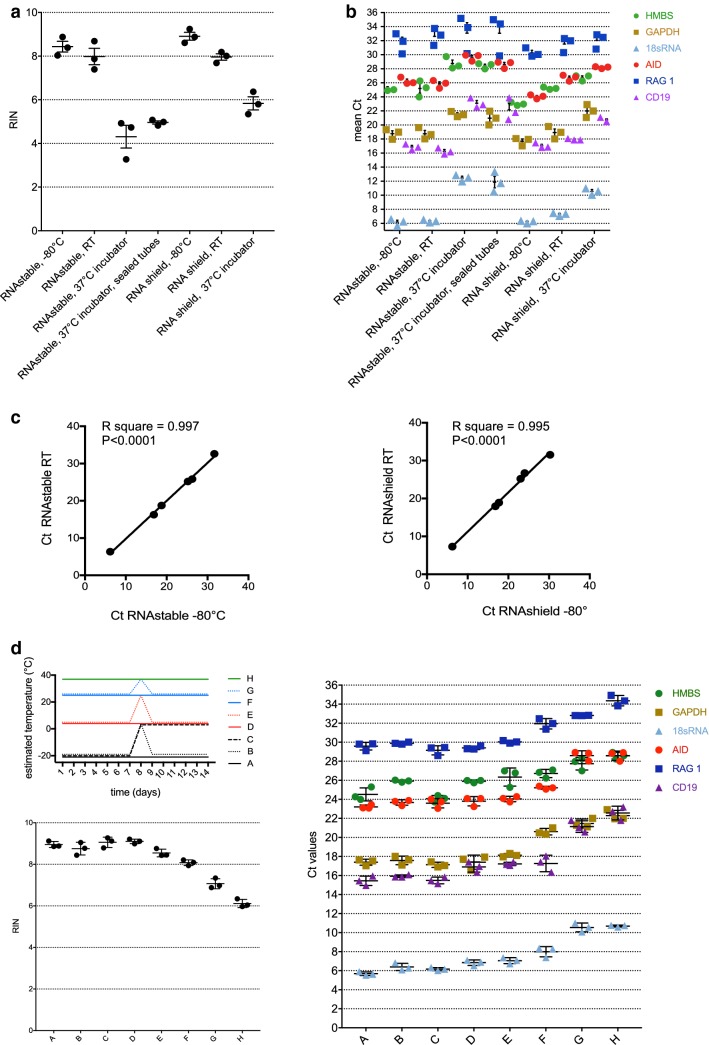
Evaluation of different preservation methods at various conditions. **a**, **b** A pellet of 10 × 10^6^ cells or RNA of 10 × 10^6^ cells, respectively, was stored as indicated (x-axis) for 1 month. **a** RIN were determined for every condition. Shown are individual data points, mean and SD of three experiments. **b** Ct values (y-axis) of three housekeeping genes and three B cell genes were measured by qRT-PCR for every condition. Shown are individual data points, mean and SD of three experiments. **c** Mean Ct values of three housekeeping genes and three B cell genes measured in samples stored in RNAstable (left panel) or RNAshield (right panel) at − 80 °C were correlated to the mean Ct values of the same genes stored at RT. **d** *Upper left panel:* schematic representation of sample storage and simulation of transient power disruption with corresponding estimated temperatures: samples were stored in RNAshield at either − 20 °C (black), 4 °C (red), 25 °C (blue) or 37 °C (green). At day 7, a power disruption was simulated for some of the samples (dotted lines) but not for other (solid lines), and at day 8, samples were placed back to their original storage temperature, except for sample **c** (dashed line). *Lower left panel:* RIN were determined for every condition. Shown are individual data points, mean and SD of three experiments. *Right panel:* Ct values (y-axis) of three housekeeping genes and three B cell genes were determined by qRT-PCR for every condition. Shown are individual data points, mean and SEM of three experiments

In the absence of any storage reagent, RNA is degraded rapidly [23]. Storage of fixed whole blood is less delicate, but after fixation, isolation of B and Th cells is no longer possible, thus gene expression cannot be attributed to a specific cell type anymore. To select a sample storage method independent of continuous cold chains that ensures RNA quality comparable to storage at − 80 °C, two different approaches were tested at three different conditions: on one side, RNA was isolated from cell pellets and subsequently dehydrated using RNAstable^®^ (Biomatrica), a reagent for stabilizing and protecting samples from degradation based on anhydrobiosis [24]. On the other side, the cell pellet was lysed in DNA/RNA shield™ (Zymo research) and stored until RNA isolation [25]. In more detail, RNA derived from 10 × 10^6^ RS4; 11 cells in RNAstable^®^ and lysate of 10 × 10^6^ RS4; 11 cells in DNA/RNA shield™ were stored for 1 month at (A) − 80 °C, (B) RT, and (C) at 37 °C with a humidity of about 95% (Fig. 4). RNA quality was assessed by determination of the RNA Integrity Number (RIN) (Fig. 4a) as well as by qRT-PCR analysis of three housekeeping genes and three B cell genes known to be expressed at different levels (Fig. 4b). Both methods to examine RNA quality revealed that RNA quality was clearly inferior when RNA was stored for 1 month at 37 °C as compared to storage at − 80 °C or RT, regardless of the preservation method (two-way ANOVA, all p values < 0.0001). Storage at RT resulted in slightly but not significantly lower RIN compared to storage at − 80 °C. In line with this, no significant changes in Ct values were observed between these two temperatures, which was consistent for all six genes analysed (Fig. 4c). Importantly, however, at 37 °C with high humidity, the DNA/RNA shield™ protected the RNA slightly but consistently better from degradation than the RNAstable^®^ (Fig. 4a, b). This may be due to the fact that the humidity lead to a partial rehydration of the RNA. Even with increased efforts of keeping the samples dry in the incubator (adding desiccant packs and sealing the tubes with paraffin film), RNA samples in RNAstable^®^ kept at 37 °C in the incubator displayed a higher degree of RNA degradation than the cell samples lysed in DNA/RNA shield™.

Rural health centres often experience power cuts, which may lead to a temporal increase in storage temperatures even in the presence of freezers, fridges or air condition. Similarly, samples may suffer from a transient increase in temperature during transportation. To address the impact of such short-term temperature changes, RNA samples stored in RNAshield for 7 days were incubated at different temperatures and a 1-day power disruption simulated, followed by determination of RIN and Ct values of six genes (Fig. 4d). RNA of samples stored at − 80 °C showed only a slight decrease in integrity when brought to 4 °C, irrespective of whether the RNA was refrozen afterwards or kept unfrozen at 4 °C for 7 days. A similar RNA quality was observed for samples stored continuously at 4 °C, warmed-up for a short period to RT or kept at RT for the whole period of 14 days. Importantly, however, short-term warm-up to 37 °C clearly deteriorated the RNA integrity, even though not as dramatically as when the samples were stored at 37 °C for 14 days.

Together, this suggests that freezers are not absolutely required for medium term sample storage, but samples are preferably stored in a fridge or a in an air-conditioned room when the environmental temperatures are above 25 °C. Most importantly, in warm and humid environments, preservation of the cell pellet in DNA/RNA shield™ is preferred over dehydration of RNA using RNAstable^®^.

### Expression of AID, IL-4 and TGF-b in B and Th cells from malaria patients

**Fig. 5:**
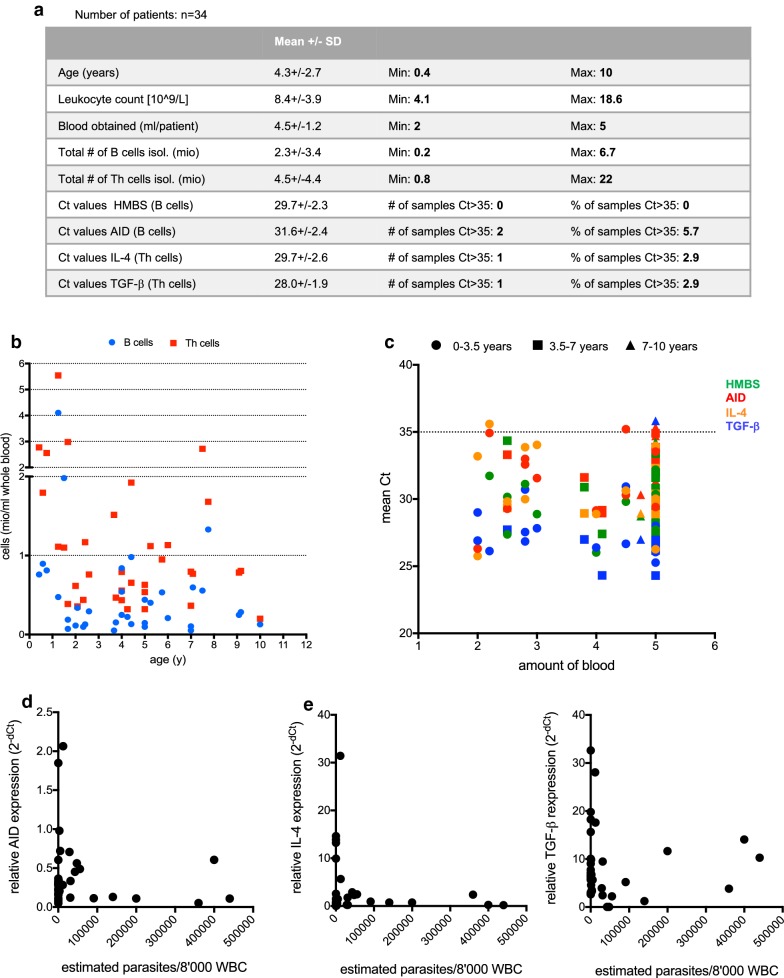
Expression of AID, IL-4 and TGF-b in B and Th cells from malaria patients. Blood was drawn from 34 patients diagnosed with malaria, B and Th cells were isolated and gene expression of AID, IL-4, TGF-b as well as the housekeeping gene HMBS was analysed by qRT-PCR. **a** Table summarizing parameters related to donors, B and Th cell isolation and gene expression analysis. **b** Age of the patients versus number of cells isolated. **c** Amount of blood withdrawn versus Ct values of HMBS (green), AID (red), IL-4 (orange) and TGF-b (blue). Symbol shapes indicate three different age groups. **d** Correlation of AID expression in B cells with parasite load. **e** Correlation of IL-4 and TGF-b expression in Th cells with parasite load

Finally, the protocol established above was used to investigate possible reasons for the impaired antibody response in malaria. Given the key role of AID in the generation of high affinity antibodies, it may be hypothesised that at a high parasite count, up-regulation of AID is inhibited, which in turn leads to a defective antibody response. To address whether malaria patients with high parasite count express lower levels of AID than those with a low count, B cells and Th cells were isolated from malaria patients and stored in RNAshield for up to 1 month at − 20 °C. Then, samples were shipped from Kitgum to Kampala (approximately 12 h in cooling bags), stored for 48 h at 4 °C and finally shipped to Switzerland (approximately 20 h with cooling bags), where RNA was isolated and gene expression was analysed by qRT-PCR (Fig. 5). Tandem B and Th cell isolation from 2 to 5 ml of blood as established above yielded 0.2–6.7 mio B cells (2.3 ± 3.4) and 0.8–22 mio Th cells (4.5 ± 4.4, Fig. 5a). As previously reported [26], younger children had more B and Th cells (Fig. 5b). With the exception of two B cells samples, Ct values were below 35, suggesting that the isolation and storage method described above was very well suited for analysis of AID expression in B cells isolated from a small amount of blood (Fig. 5a, c). Since TGF-b and IL-4 are the main cytokines that induce AID expression [11], their expression levels in Th cells of malaria patients were analysed. Ct values of these cytokines were well below 35 except in one sample. Then, the parasite load was correlated with expression of AID, TGF-b and IL-4 (Fig. 5d, e). Even though the highest expression of AID, IL-4 and TGF-b was indeed observed in patients with low parasite count, there was no significant correlation between parasite count and these genes.

Taken together, while no correlation between high parasite count and low AID expression was found, the protocol established here was clearly suitable for gene analysis by qRT-PCR in B cells and Th cells isolated from small amounts of blood from paediatric malaria patients.

## Discussion

This study presents a protocol for high purity B cell and Th cell isolation and medium-term sample storage for standard qRT-PCR analysis from small amounts of blood that is contrivable in basically equipped laboratories.

PBMC isolation on a density gradient requires a sophisticated centrifuge that is able to slowly and steadily increase the speed from 0 to 800 g and back to 0 g. Thus, any power cut as they frequently happen in rural areas—would cause a sudden stop of the centrifuge. As a consequence, the density gradient is disturbed and the samples are lost. By using tandem B and Th cell isolation directly from blood, the need for such a centrifuge and continuous power supply could be circumvented. Furthermore, the protocol described here is independent from ice, it is fast (1–2.5 h, depending on the amount of blood and number of magnets available), easy to learn and to perform, and it yields excellent purities. The fact that the number of isolated cells has been consistent between the experiments further demonstrates its reproducibility and robustness. One caveat of the protocol is the fact that positive selection conducted at RT may activate the cells and therefore affect gene expression. Nevertheless, the protocol is relatively fast. Thus, the time from a potential activation until the cells are lysed is short, and a substantial de novo mRNA synthesis is not expected. A further downside of the method is the cost of the magnetic beads with a price of around 44 USD for each B cell isolation from 4 ml whole blood, and a one-time cost for the magnet of 1140 USD. Still, other positive B cell isolation kits from whole blood such as the Dynabeads™ CD19 Pan B kit [27] or the Miltenyi Biotec MACSprep Chimerism CD19 MicroBeads human kit [28] reside within the same price range with 36–60 USD per isolation and a magnet cost between 1076 USD and 13,200 USD. Less expensive is negative and positive B cell selection from previously isolated PBMC with about 1.6–4.4 USD/isolation and a magnet prize between 826 and 2400 USD (e.g. MagniSort™ Human CD19 Positive Selection Kit, [20]. MojoSort™ Human Pan B Cell Isolation Kit [21], Miltenyi Biotec B Cell Isolation Kit II, human [22]). However, it has to be considered that these latter kits require strong and continuously running centrifuges, additional reagents for a density gradient, such as a Ficoll (cost per around 1.5 USD/4 ml whole blood sample), and are more time-consuming. Furthermore, funding bodies are generally more compliant to fund expenditure than equipment of remaining value.

In this study, 2–5 ml blood of malaria patients was used to analyse gene expression in B and Th cells. 4–5 ml of blood is an amount that is not negligible in little children. The results of this study show that except from one sample, even 2–3 ml blood was sufficient for reliable gene expression analysis of various genes (Fig. 5c), and higher amounts did not substantially improve the Ct values. Nevertheless, the number of B cells in the blood is highly dependent on the age of the donor and may deviate dramatically from the average in particular diseases settings, such as leukopenia in severe malaria. Thus, the amount of blood required not only depends on the genes to be investigated, but also on the donors. The results of this study reporting cell numbers and Ct values obtained from different amounts of blood from healthy and sick children of different age groups therefore provide information that can help investigators to choose an appropriate amount of blood for a given study.

While no marked differences between the two sample storage approaches were observed at − 80 °C and RT, preservation of the cell pellet in DNA/RNA shield™ was slightly but consistently superior to preservation of RNA in RNAstable^®^ at 37 °C and a high humidity, where the RNA may get rehydrated. Thus, in warm and humid environments typical for malaria regions, storage of the pellet in DNA/RNA shield™ is preferred. Furthermore, usage of DNA/RNA shield™ enables deferral of the RNA extraction to any suitable time-point, allowing for transport of the samples to a laboratory equipped for RNA isolation and further downstream analysis. Finally, it is significantly less expensive (DNA/RNA shield™ approx. 0.7 USD for 1 sample, RNAstable^®^ 2.7 USD per sample).

RNA stored in RNAshield at RT was of sufficient quality for the application of this study. Nevertheless, it needs to be considered that different RNAs degrade with different speed. Thus, even though the correlation of the Ct values between samples stored at RT and − 80 was close to 1 in the six genes analysed here, performing RNA-seq analysis may provide a more comprehensive validation of the suitability of the storage condition.

No correlation between parasite load and AID, IL-4, or TGF-b expression was observed. While it is certainly possible that these factors in fact do not correlate, a potential correlation may have been missed for technical reasons such as wrong time point of measurement, too small samples size or too few patients with high parasite load. Moreover, parasite load was only estimated by the use of light microscopy, and a more accurate method for parasite quantification such as qRT-PCR may lead to a different result.

Nevertheless, the protocol presented here for tandem B and Th cell isolation proved to be suitable for gene analysis by qRT-PCR in B cell and Th cell isolated form small amounts of blood from paediatric malaria patients.

## Conclusion

The protocol described here delineates a fast and efficient way of high purity B and Th cells isolation and storage from a minimal amount of blood in a resource limited environment. Even though isolation of lymphocytes from whole blood using magnetic beads is not a new concept in itself, an optimized and detailed protocol for tandem B and T cell isolation from as little as 2–5 ml blood has not been available so far. The excellent purity of the isolated cells, the simplicity and reproducibility of the procedure and the fact that it can be conducted without any major laboratory equipment makes it a valuable tool to investigate the role of B cells and Th cells in malaria, which ultimately may lead to the development of novel therapeutic strategies.
